# Supplementary material for: Prevalence and patterns of substance use in West Africa: A systematic review and meta-analysis
Source: PLOS Glob Public Health. 2024 Dec 31;4(12):e0004019. doi: 10.1371/journal.pgph.0004019 (PMC11687703; doi:10.1371/journal.pgph.0004019)
Supplement: S2 File — (PDF) [file pgph.0004019.s002.pdf]

Supplementary File 2_Search Terms and Strategy

CINAHL

Search strategy	Results
(“Drug addiction” OR “drug dependence” OR “substance addiction”) AND PREVALENCE<br><br>Limiters - Publication Date: 20000101-20241231; Exclude MEDLINE records; Geographic Subset: Africa<br>Expanders - Apply equivalent subjects<br>Search modes - Proximity Interface - EBSCOhost Research Databases<br>Search Screen - Advanced Search<br>Database - CINAHL Complete	15

PubMed

	Key words	Search strategy	Results
#1	Drug addiction	(("substance addiction"[Title/Abstract]) OR ("drug dependence"[Title/Abstract])) OR ("Drug addiction"[Title/Abstract])	13,869
#2	Prevalence	PREVALENCE[MeSH Terms]	354,671
#3	#1 AND #2	((("substance addiction"[Title/Abstract]) OR ("drug dependence"[Title/Abstract])) OR ("Drug addiction"[Title/Abstract])) AND (PREVALENCE[MeSH Terms])	461
#4	Filter by year	((("substance addiction"[Title/Abstract]) OR ("drug dependence"[Title/Abstract])) OR ("Drug addiction"[Title/Abstract])) AND (PREVALENCE[MeSH Terms]) Filters: from 2000 - 2024	367

WEB OF SCIENCE

	Key words	Search strategy	Results
#1	Drug addiction	((TS=(Drug addiction*)) OR TS=(drug dependence*)) OR TS=(“substance addiction”) and Preprint Citation Index (Exclude – Database)	341,409
#2	Prevalence	TS=(Prevalence) and Preprint Citation Index (Exclude – Database)	1,667,277
#3	#1 AND #2	#1 AND #2 and Preprint Citation Index (Exclude – Database)	30,170
#4	FILTER	#1 AND #2 and Preprint Citation Index (Exclude – Database) and 2024 or 2023 or 2022 or 2021 or 2020 or 2019 or 2018 or 2017 or 2016 or 2015 or 2014 or 2013 or 2012 or 2011 or 2010 or 2009 or 2008 or 2007 or 2006 or 2005 or 2004 or	142

		2003 or 2002 or 2001 or 2000 (Publication Years) and Article (Document Types) and Web of Science Core Collection (Database) and BENIN or BURKINA FASO or CAPE VERDE or GAMBIA or GHANA or GUINEA or MALI or MAURITANIA or NIGERIA or SENEGAL or SIERRA LEONE (Countries/Regions)	

Scopus

Search strategy	Results
(TITLE-ABS-KEY ("drug addiction" OR "drug dependence" OR "substance addiction") AND TITLE-ABS-KEY (prevalence)) AND PUBYEAR > 1999 AND PUBYEAR < 2025 AND (LIMIT-TO (AFFILCOUNTRY , "nigeria") OR LIMIT-TO (AFFILCOUNTRY , "ghana") OR LIMIT-TO (AFFILCOUNTRY , "benin") OR LIMIT-TO (AFFILCOUNTRY , "liberia") OR LIMIT-TO (AFFILCOUNTRY , "senegal") OR LIMIT-TO (AFFILCOUNTRY , "burkina faso") OR LIMIT-TO (AFFILCOUNTRY , "mali") OR LIMIT-TO (AFFILCOUNTRY , "togo"))	88
