# Supplementary material for: Prevalence and patterns of substance use in West Africa: A systematic review and meta-analysis
Source: PLOS Glob Public Health. 2024 Dec 31;4(12):e0004019. doi: 10.1371/journal.pgph.0004019 (PMC11687703; doi:10.1371/journal.pgph.0004019)
Supplement: S3 File — (PDF) [file pgph.0004019.s003.pdf]

Supplementary File 3: Excluded studies with Reasons

S/No	Study ID	Title	Reasons for Exclusion
1.	Krippler and Kittel, 2010	Drug addiction in the working environment: Prevalence of use of psychoactive substances use and its relationship to high-risk occupation and stress	The study focuses on a specific population of employees in Luxembourg, and our study focuses on West African populations.
2.	Onaolapo et al. 2022	Substance use and substance use disorders in Africa: An epidemiological approach to the review of existing literature	The study is a narrative review rather than a primary research article. Our review focuses on primary data studies, including cohort studies, cross-sectional studies, or randomized controlled trials, that provide original data on substance use patterns and prevalence. As this study is a secondary review of existing literature, it does not meet the inclusion criteria.
3.	Forson et al., 2020	Emergency department admissions Kumasi, Ghana: Prevalence of alcohol and substance use, and associated trauma	The study focuses on emergency department admissions related to trauma and altered mental status.
4.	Akinbami et al. 2019	Pentazocine Addiction among Sickle Cell Disease Patients and Perception of Its Use among Health-Care Workers	The study focuses specifically on sickle cell disease (SCD) patients, which differs from the broader population we aimed to study in our systematic review. Our review focuses on general substance use patterns across wider populations, not specific patient groups like those with chronic diseases such as SCD.
5.	Uthman et al. 2022	Psychoactive substance use disorders in an adult sickle cell disease population in Nigeria: prevalence and correlates	This study focuses exclusively on individuals with sickle cell disease (SCD). Our review is focused on general population-based data concerning substance use, without an emphasis on specific health conditions or populations like SCD patients.<br>The study's primary outcome relates to the prevalence and correlates of substance use disorder (SUD) in an SCD cohort, with a significant focus on factors such as social support and stigma. This differs from our review's focus on the broader patterns of substance use in a general population context.
6.	Chia et al. 2023	Access to drug treatment services in Nigeria: the challenge of the addiction workforce	The study focuses on addiction workforce as a factor with the highest weight in affecting access to drug treatment services in Nigeria
7.	Okoro et al. 2024	Prevalence of alcohol and other psychoactive substance abuse and association with depression among medical students in Niger Delta University, Bayelsa State	The primary focus of the study was on the association between substance use and depression. In contrast, our review focuses on the general prevalence and patterns of substance use without a specific emphasis on its relationship with mental health conditions such as depression.
8.	Abubakar et al. 2021	Knowledge of health effects and determinants of psychoactive substance use among secondary school students in Sokoto Metropolis, Nigeria	This study focuses primarily on knowledge and determinants of psychoactive substance use among secondary school students, which may differ from the specific outcomes of interest in your review, such as patterns of substance use or clinical outcomes in different populations.
9.	Onifade et al. 2011	A descriptive survey of types, spread and characteristics of substance abuse treatment centers in Nigeria	This study focused on treatment centers rather than individual users.
10.	Dapap et al. 2020	Use of psychoactive substances among patients presenting at the emergency department of a tertiary hospital	This study determined the prevalence and pattern of psychoactive substance use among patients presenting in the Accident and EDs and to compare the case detection rate of psychoactive substance use between self-report questionnaire and biochemical markers.
