# Supplementary material for: Prevalence and patterns of substance use in West Africa: A systematic review and meta-analysis
Source: PLOS Glob Public Health. 2024 Dec 31;4(12):e0004019. doi: 10.1371/journal.pgph.0004019 (PMC11687703; doi:10.1371/journal.pgph.0004019)
Supplement: S6 File — (PDF) [file pgph.0004019.s006.pdf]

Supplementary File 5: Quality and Risk of Bias Assessment

S/N	Author's/Year	Was the sample frame appropriate to address the target population?	Were study participants sampled in an appropriate way?	Was the sample size adequate?	Were the study subjects and the setting described in detail?	Was the data analysis conducted with sufficient coverage of the identified sample?	Were valid methods used for the identification of the condition?	Was the condition measured in a standard, reliable way for all participants?	Was there appropriate statistical analysis?	Was the response rate adequate, and if yes, was the low response rate managed appropriately?	Total score	Grade
1	Abdulkarim et al., (2005) (22)	0	0	0	0	0	1	1	0	0	2	Low
2	Adebowale & James, (2018) (23)	0	0	0	0	0	0	0	0	0	0	Low
3	Aigbogun et al., (2024) (24)	0	0	0	0	0	0	0	0	0	0	Low
4	Aluh et al., (2023) (25)	1	0	0	0	0	0	0	1	0	2	Low
5	Amoah et al., (2022) (26)	0	0	0	0	0	1	0	0	0	1	Low
6	Anyanwu et al., (2017) (27)	0	0	0	0	0	1	0	0	0	1	Low
7	Asante & Atorkey, (2023) (28)	0	0	0	0	0	1	0	0	0	1	Low
8	Bio-sya et al., (2022) (34)	0	0	0	0	0	0	0	0	0	0	Low
9	Danso & Anto, (2021) (35)	0	0	0	0	0	0	0	0	0	0	Low
10	Forson et al., (2020) (36)	0	1	0	0	1	1	1	1	0	5	Moderate
11	Gureje et al., (2007) (37)	0	0	0	0	0	0	0	0	0	0	Low
12	Idowu et al., (2018) (38)	0	0	0	0	0	0	0	0	0	0	Low
13	Kyei-Gyamfi et al., (2024) (13)	0	0	0	0	0	1	1	0	0	2	Low
14	Lasebikan & Ijomanta, (2018) (39)	0	0	0	0	1	0	0	0	0	1	Low
15	Makanjuola et al., (2010) (40)	0	0	0	0	0	1	0	0	0	1	Low
16	Mirian Aguocha & Nwefoh, (2021) (41)	0	0	0	0	1	0	1	0	0	2	Low

17	Obadeji et al., (2020) (42)	0	0	0	0	0	0	0	0	0	0	Low
18	Odukoya et al., (2018) (29)	0	0	0	0	0	0	0	0	0	0	Low
19	Olanrewaju et al., (2022) (30)	0	0	0	0	1	0	0	0	0	1	Low
20	Soremekun et al., (2020) (31)	0	0	0	0	0	0	0	0	0	0	Low
21	Umar et al., (2017) (32)	0	0	0	0	0	0	0	0	0	0	Low
22	Vigna-Taglianti et al., (2019) (33)	0	0	0	0	0	0	0	0	0	0	Low
