# Supplementary figures and images for: Prevalence and patterns of substance use in West Africa: A systematic review and meta-analysis
Source: PLOS Glob Public Health. 2024 Dec 31;4(12):e0004019. doi: 10.1371/journal.pgph.0004019 (PMC11687703; doi:10.1371/journal.pgph.0004019)

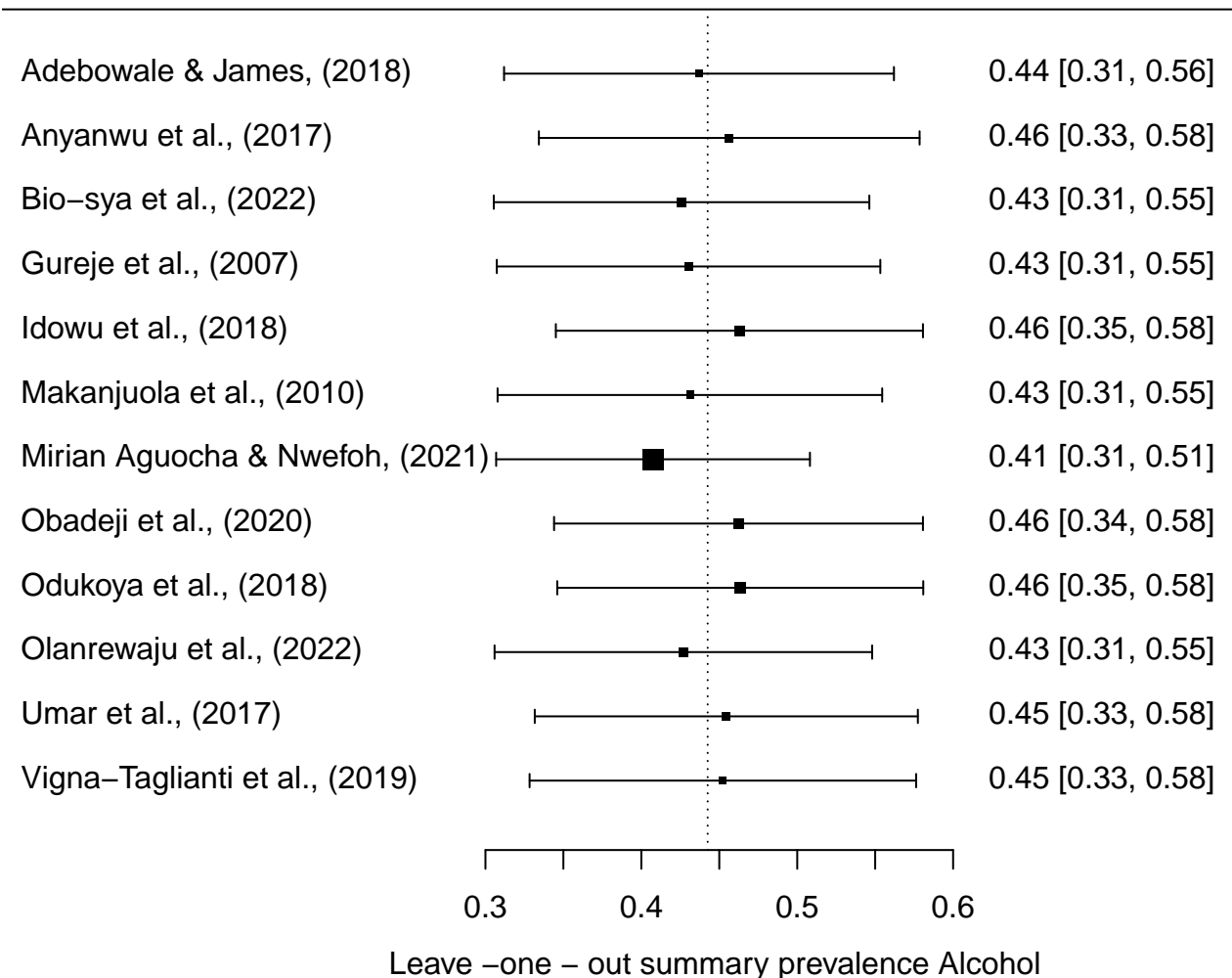

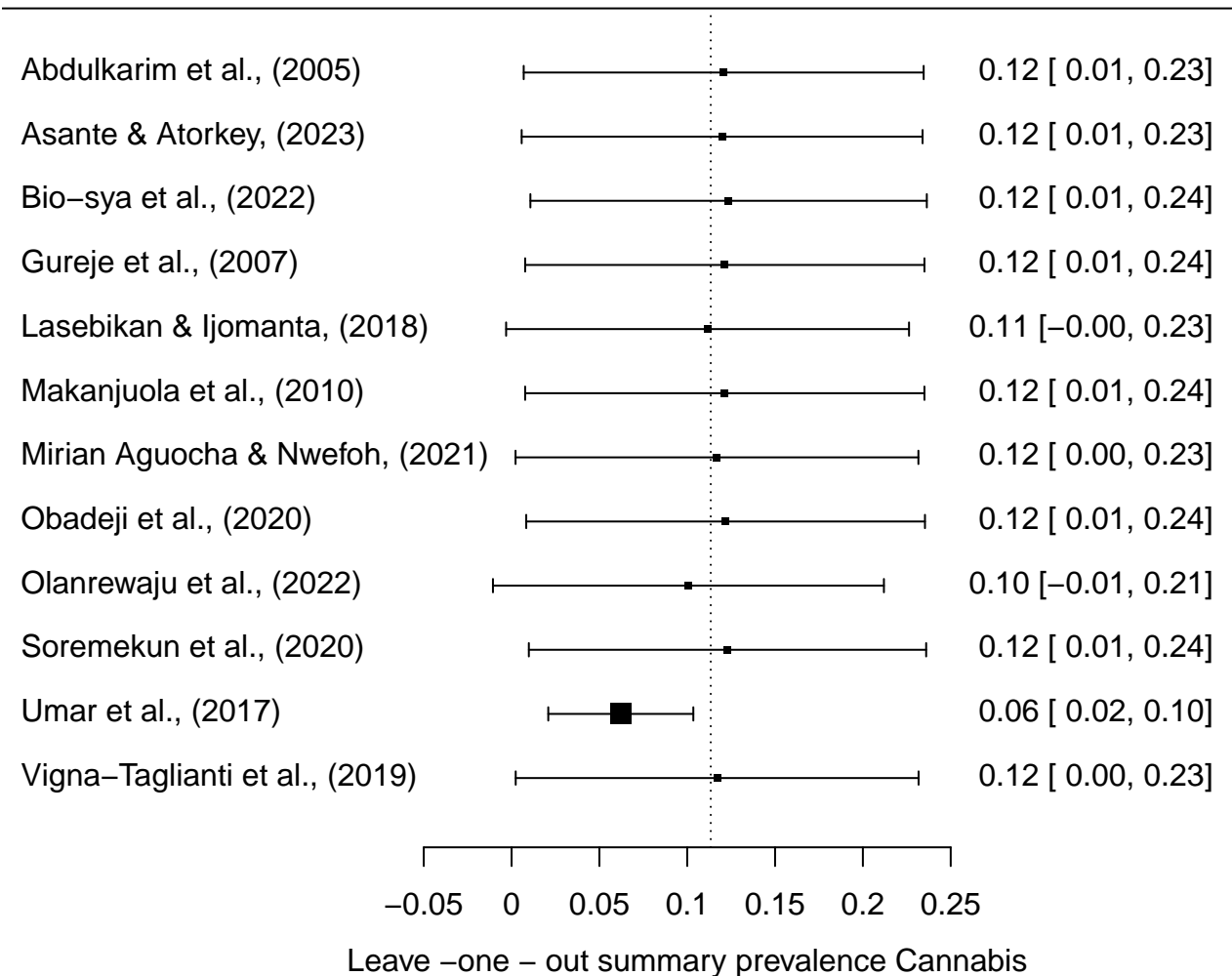

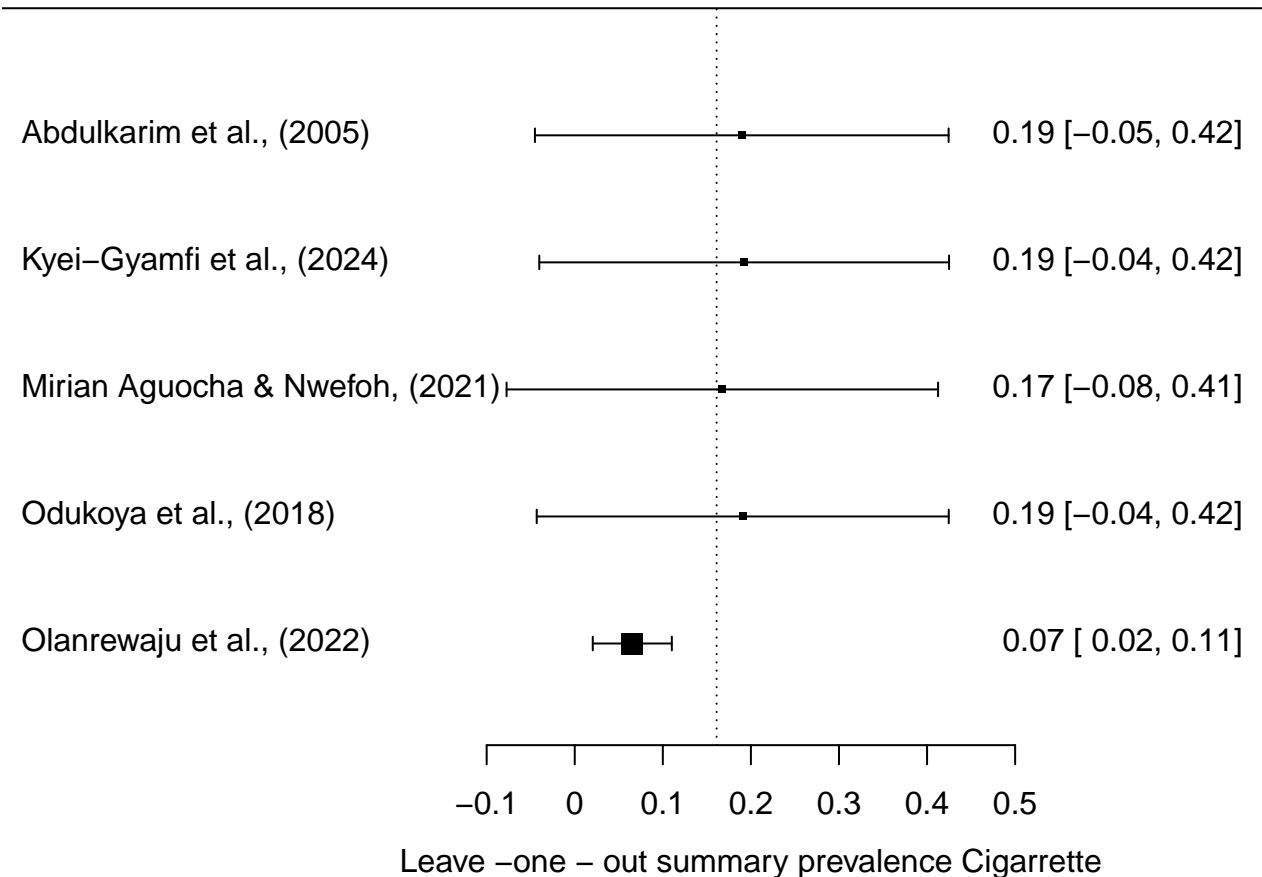

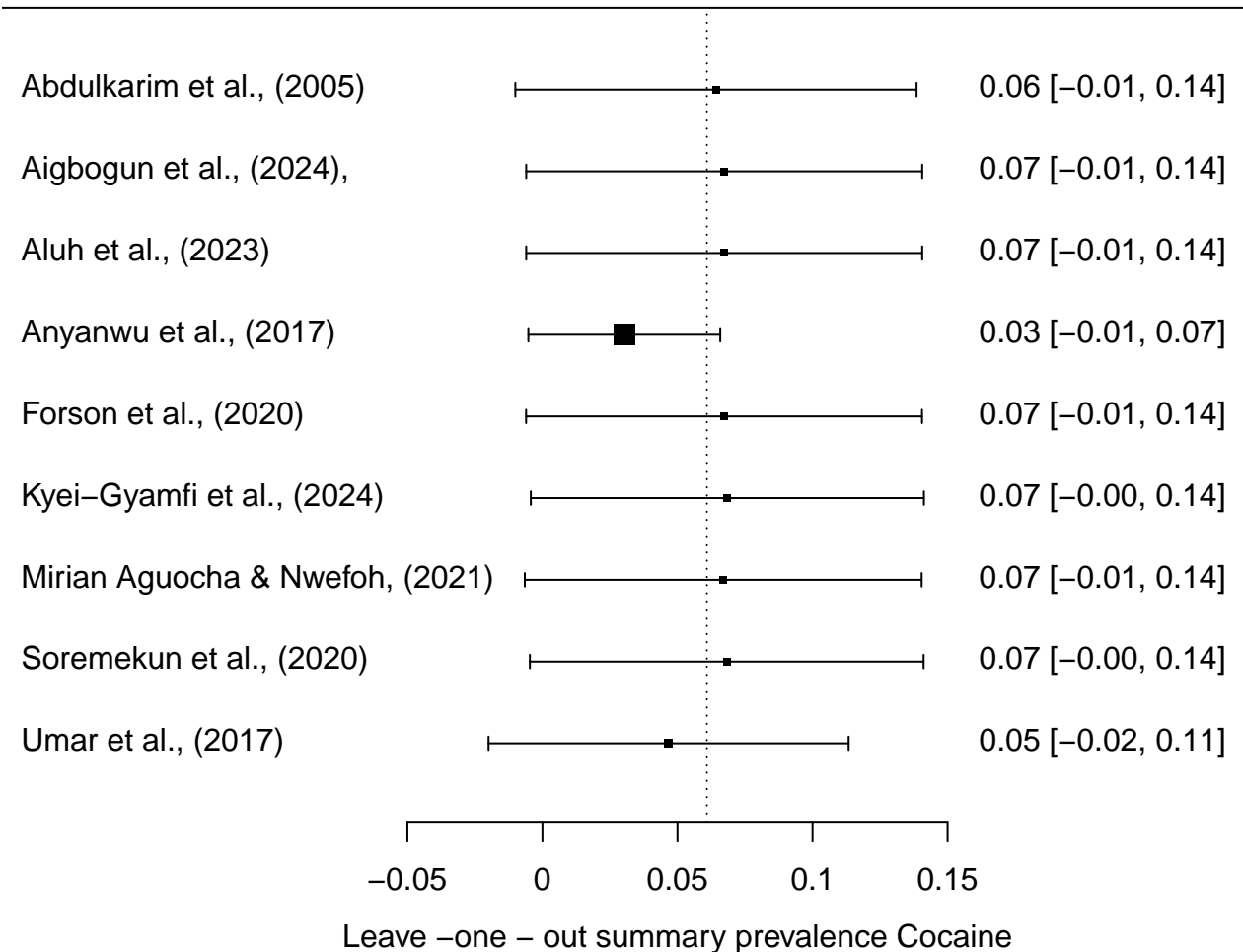

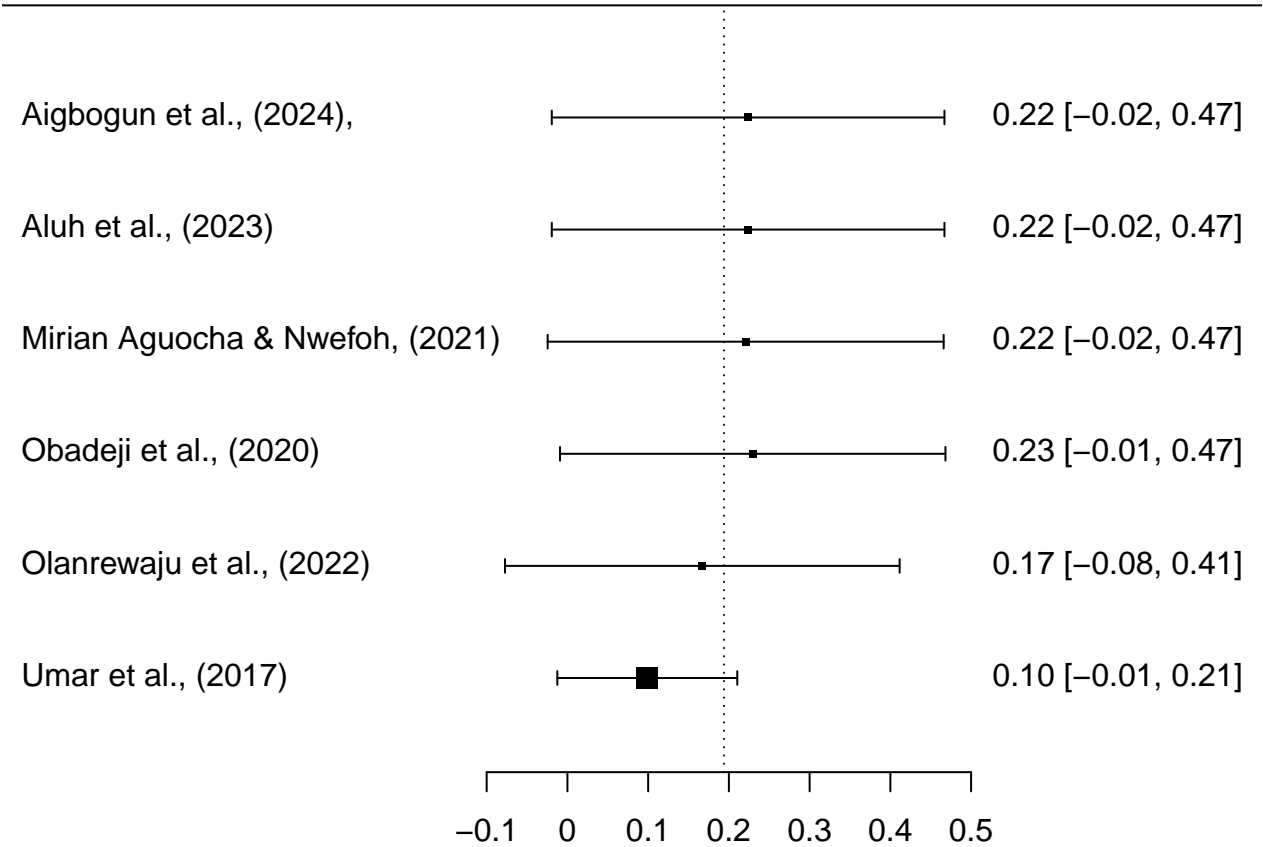

Leave –one – out summary prevalence Codeine

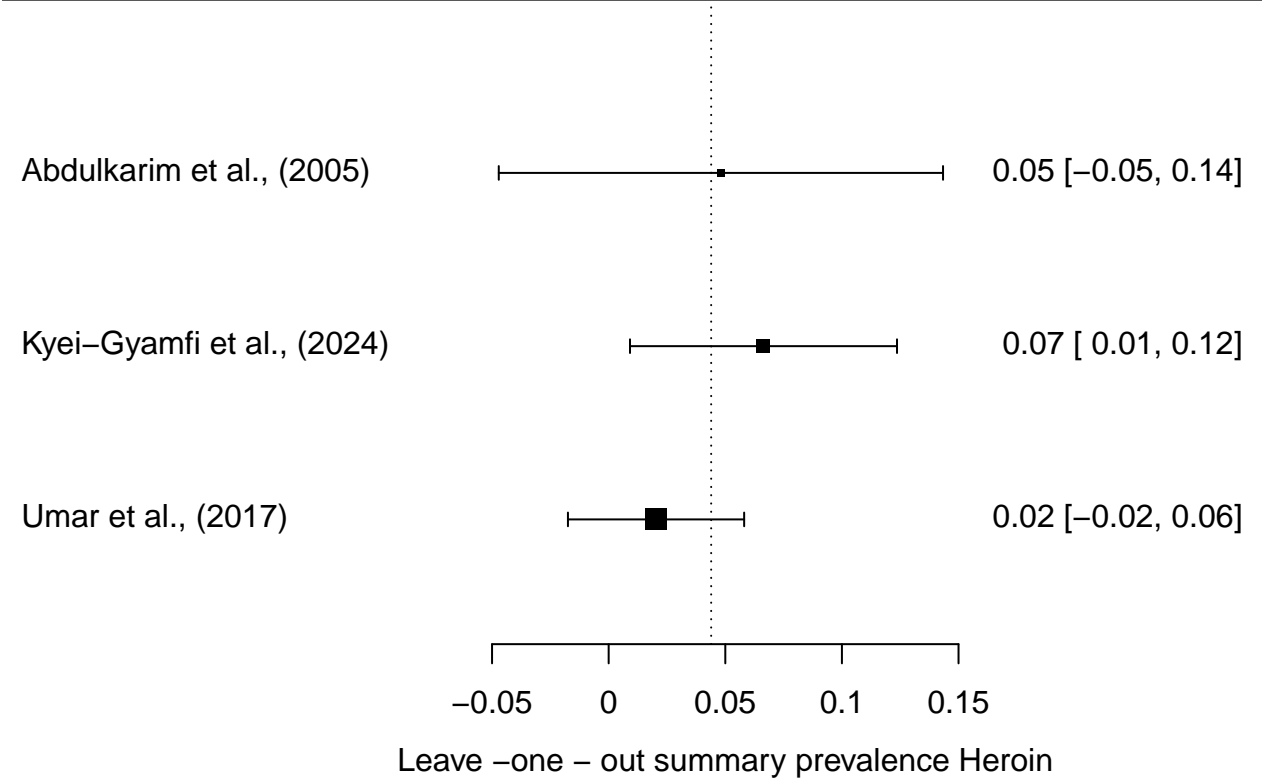

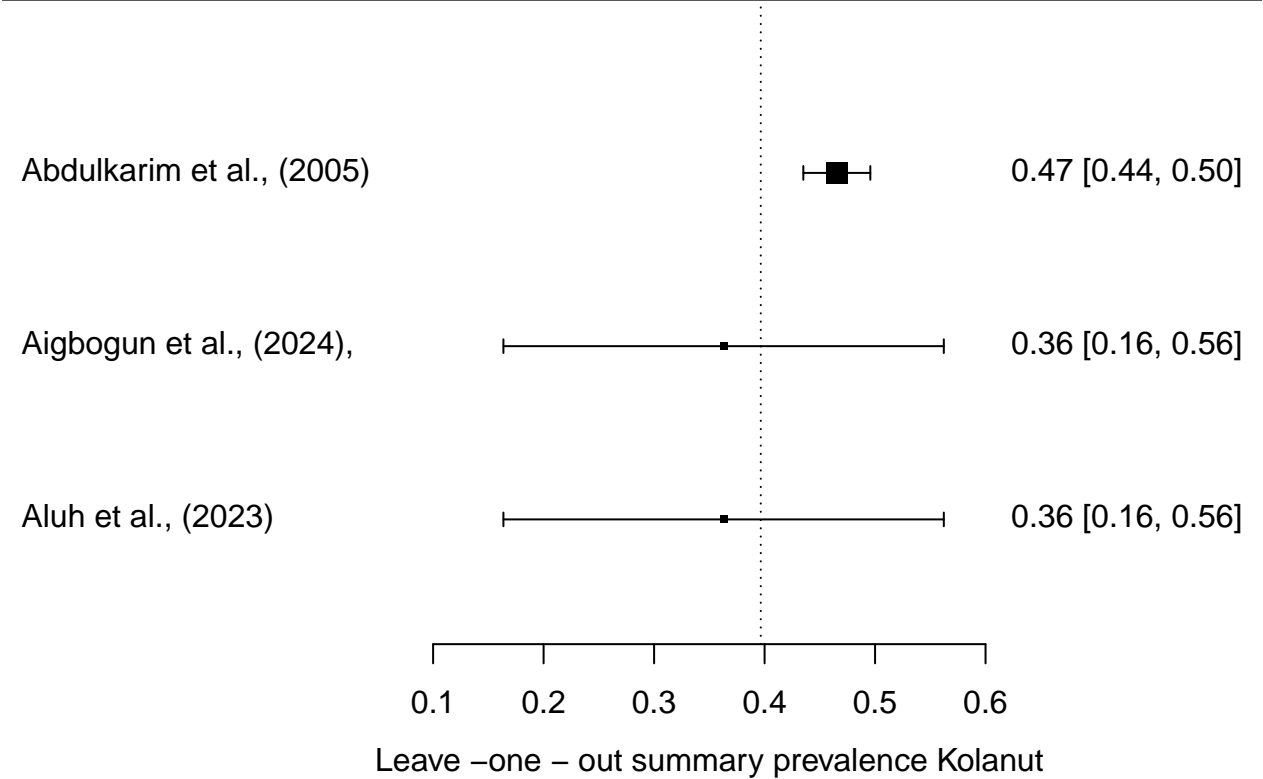

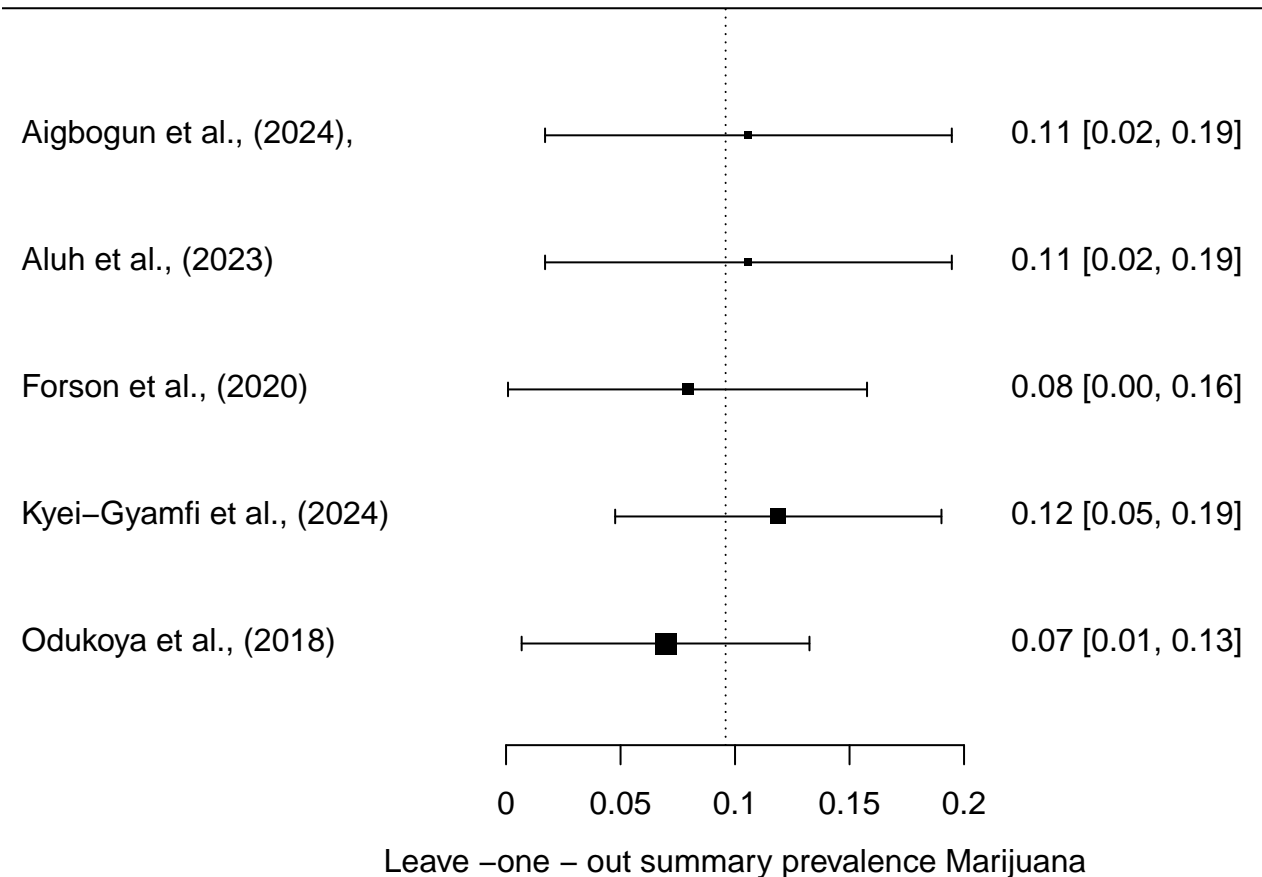

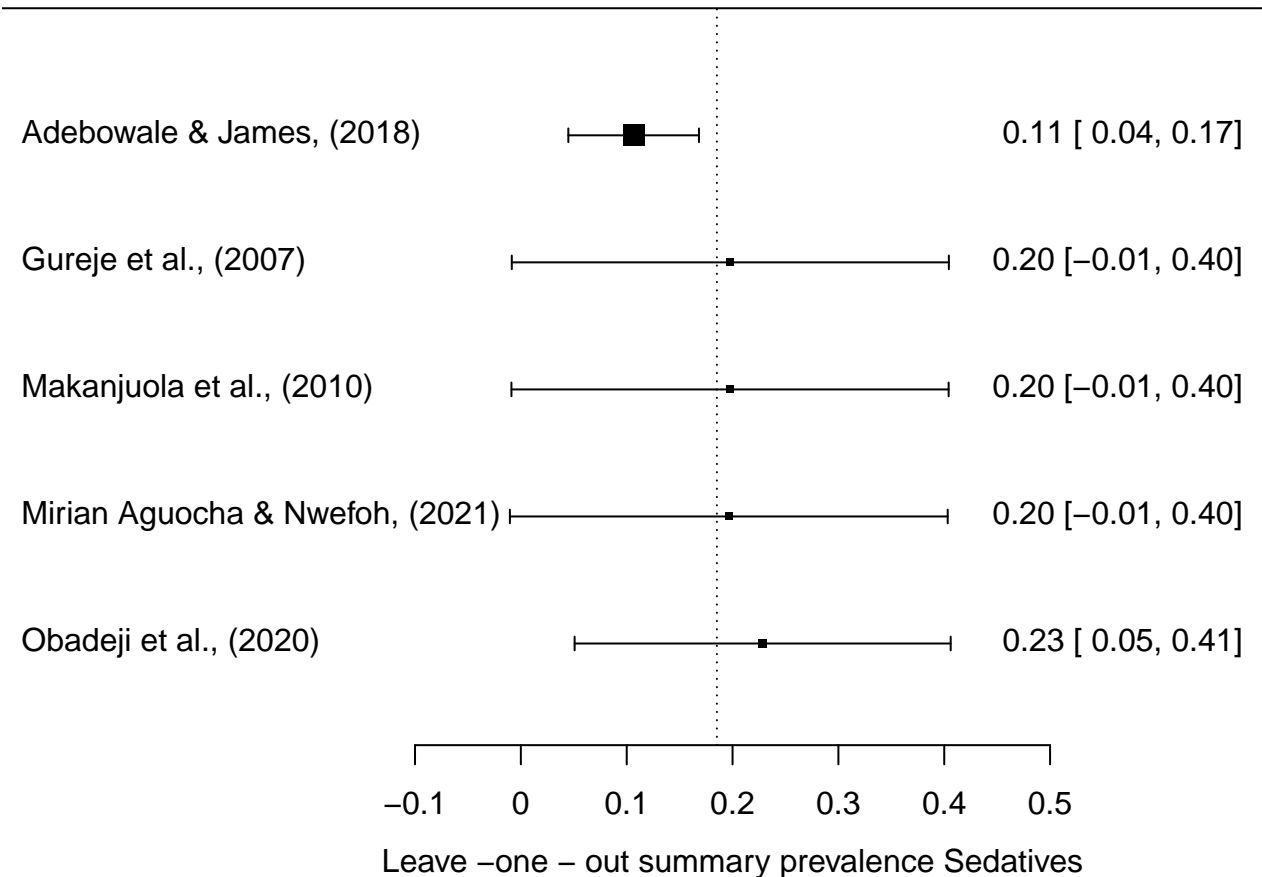

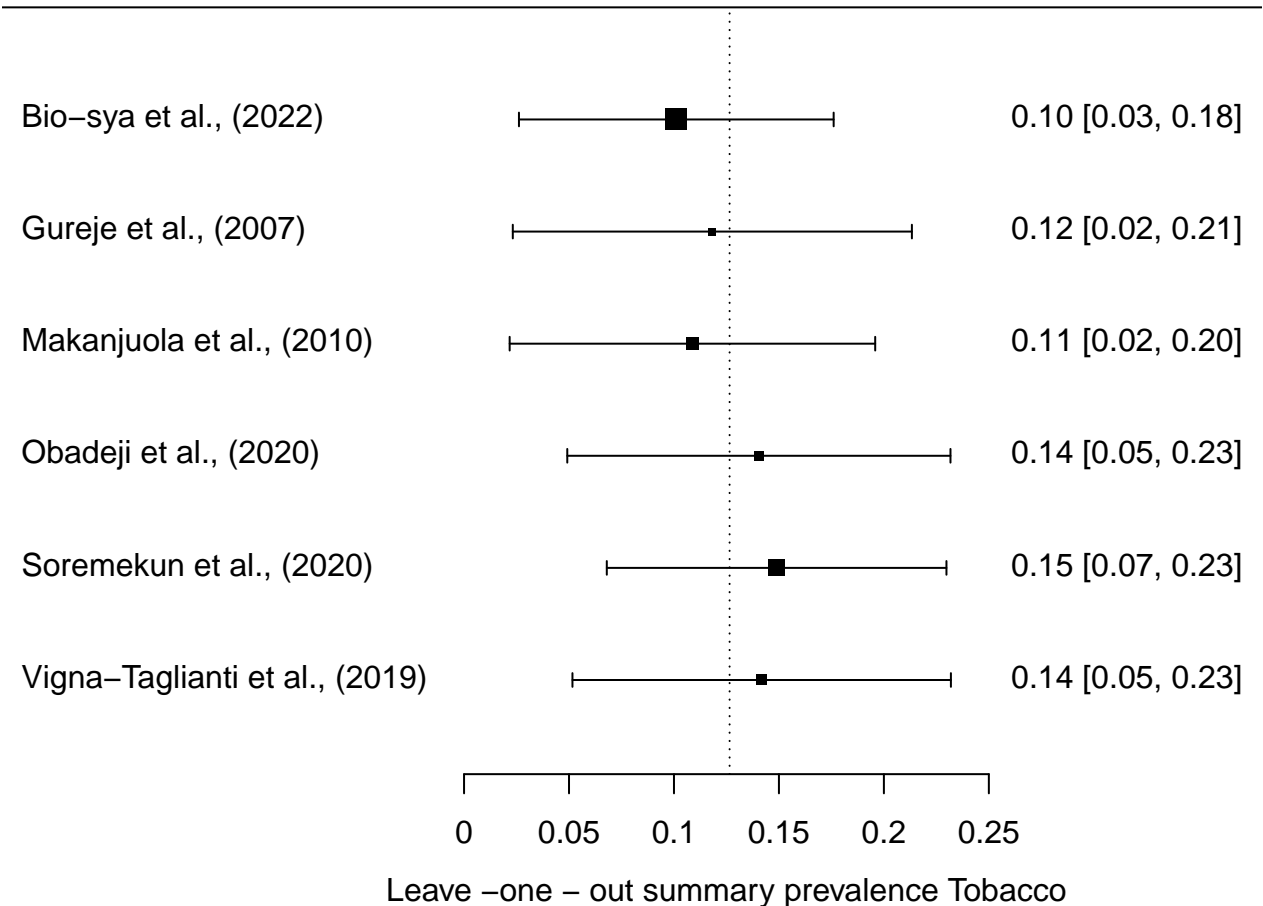

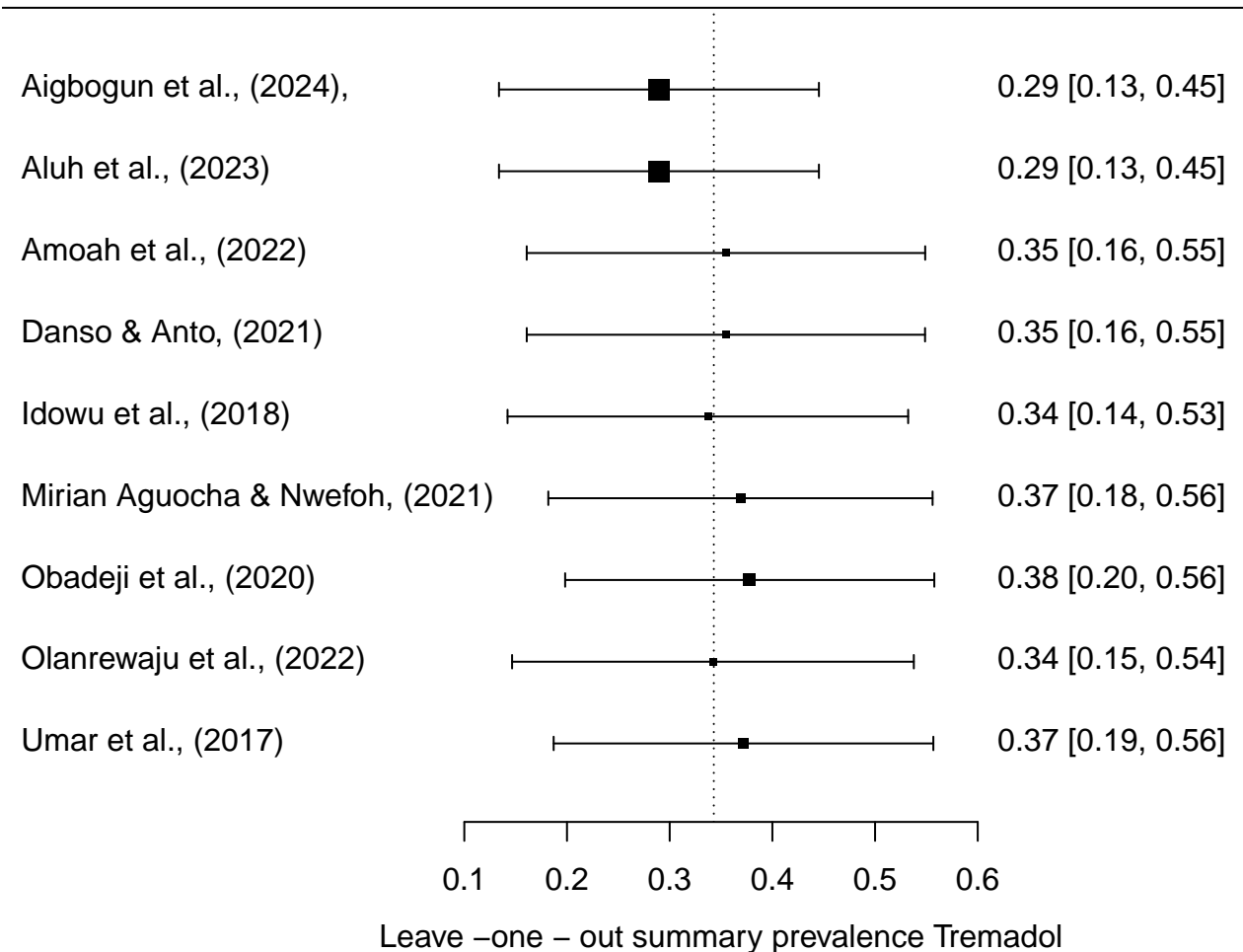

Supplement: S7 File — (PDF) [file pgph.0004019.s007.pdf]

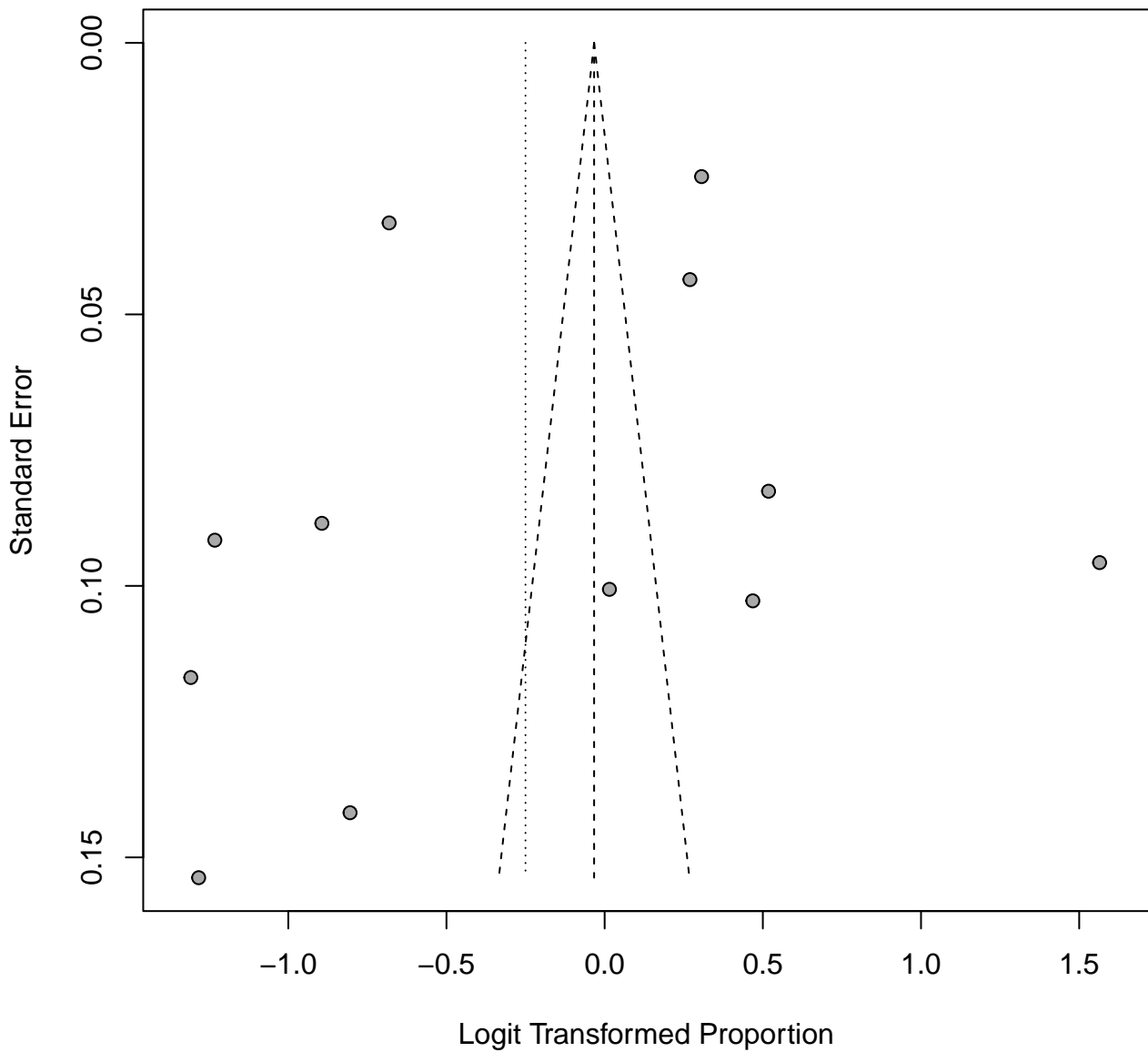

Supplement: S8 File — (ZIP) [file pgph.0004019.s008.zip › Supplementary File 4_Funnel Plots/Alcohol_funnel.pdf]

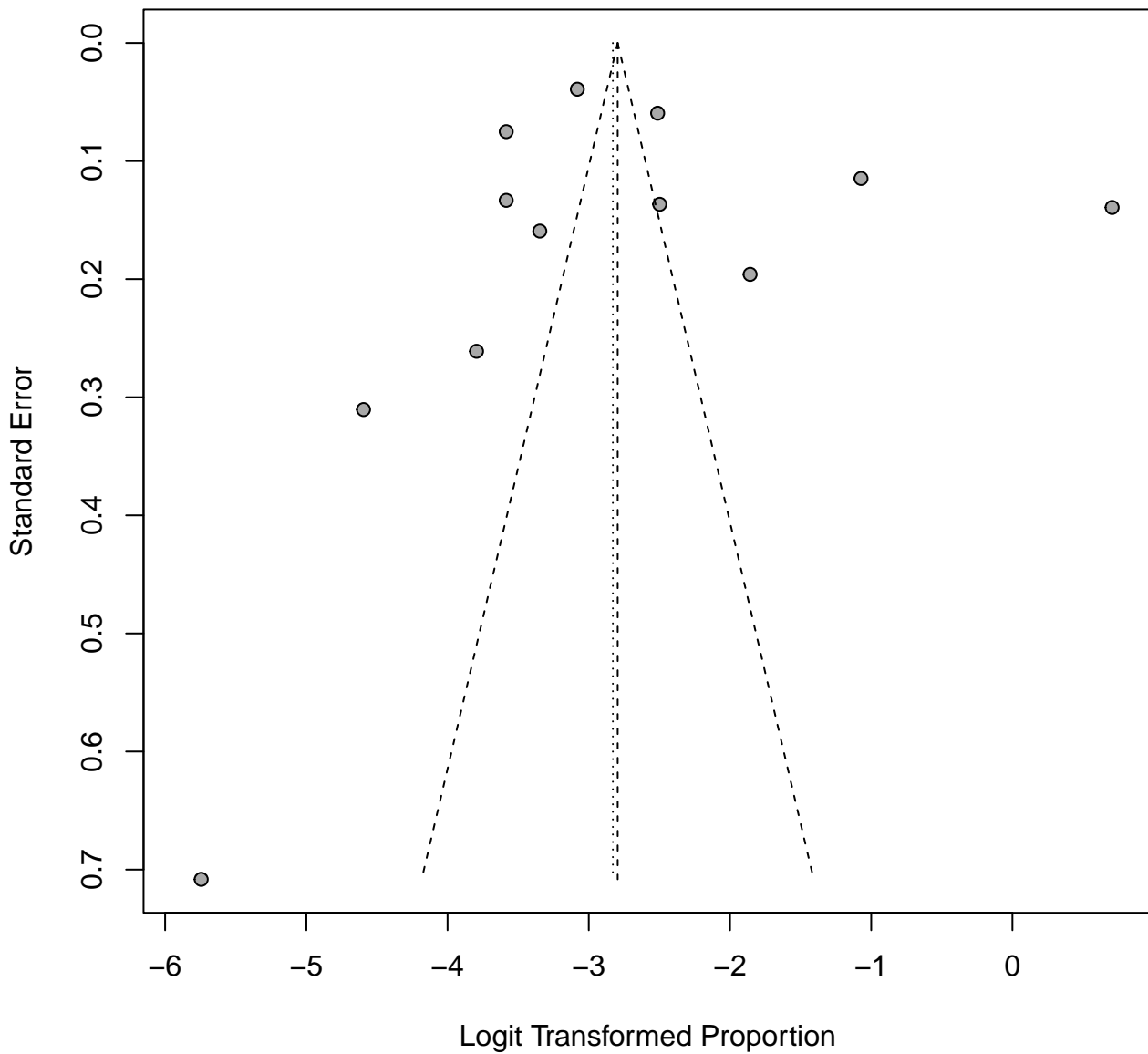

Supplement: S8 File — (ZIP) [file pgph.0004019.s008.zip › Supplementary File 4_Funnel Plots/Cannabis_funnel.pdf]

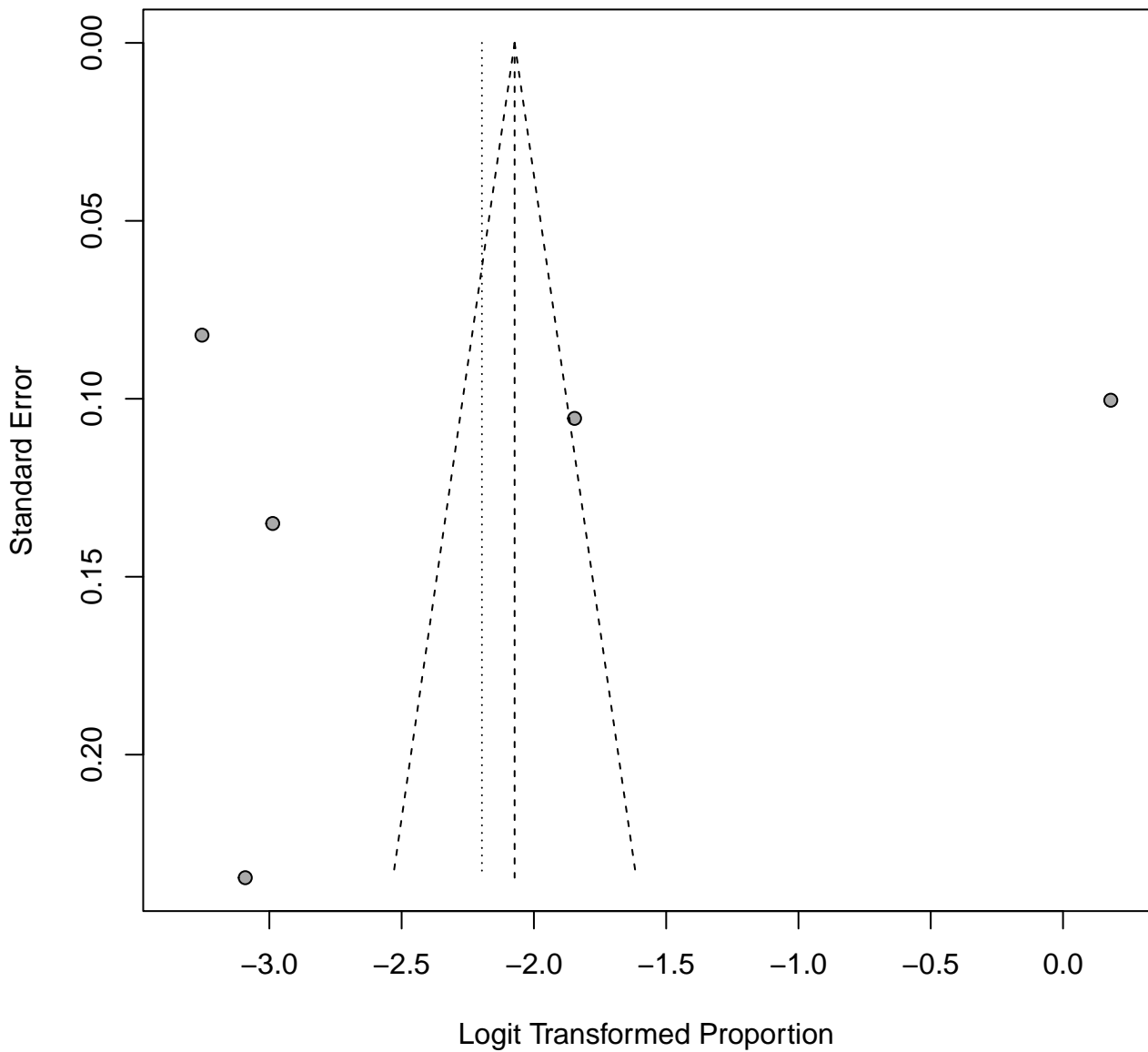

Supplement: S8 File — (ZIP) [file pgph.0004019.s008.zip › Supplementary File 4_Funnel Plots/Cigarrette_funnel.pdf]

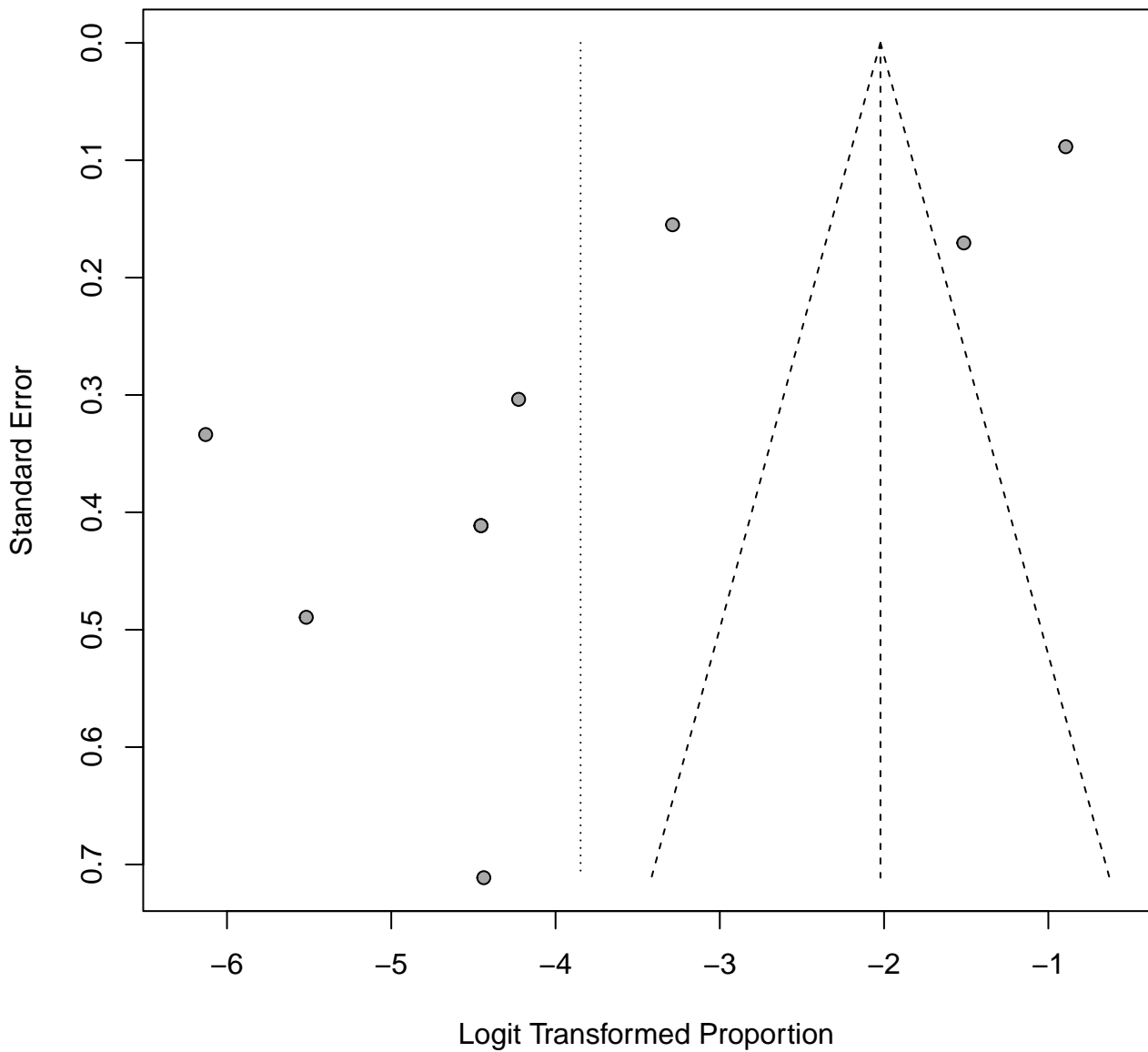

Supplement: S8 File — (ZIP) [file pgph.0004019.s008.zip › Supplementary File 4_Funnel Plots/Cocaine_funnel.pdf]

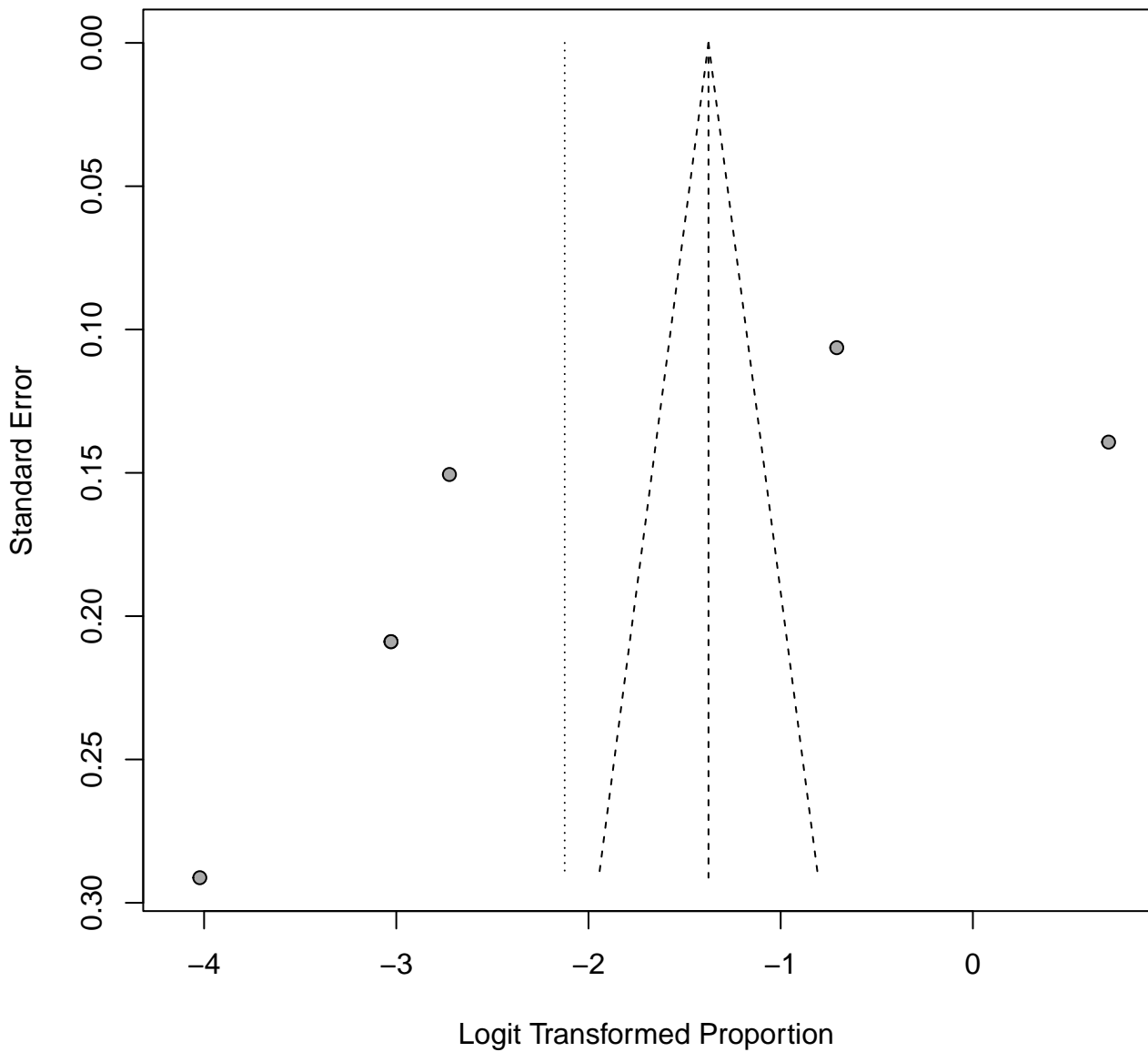

Supplement: S8 File — (ZIP) [file pgph.0004019.s008.zip › Supplementary File 4_Funnel Plots/Codeine_funnel.pdf]

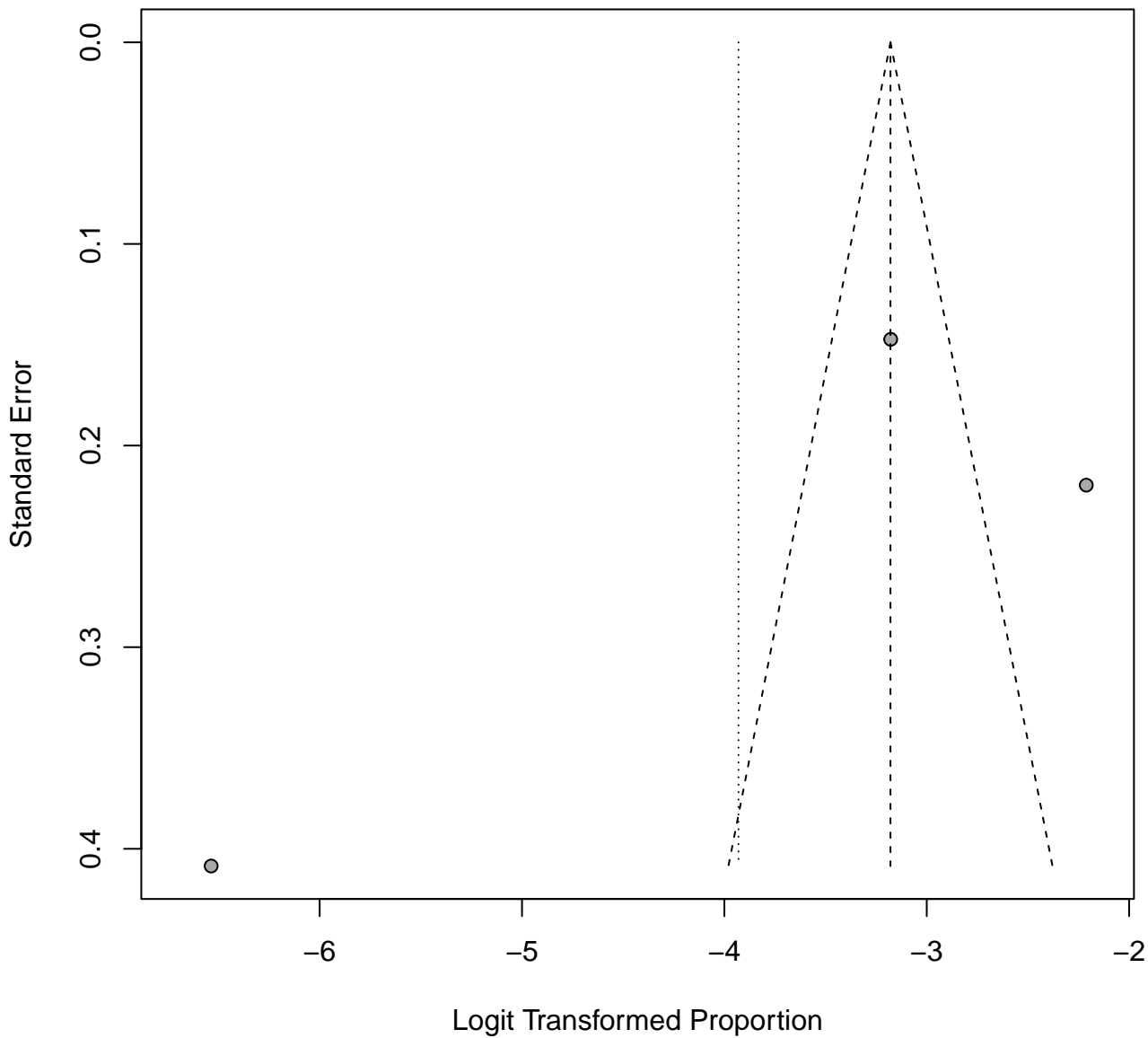

Supplement: S8 File — (ZIP) [file pgph.0004019.s008.zip › Supplementary File 4_Funnel Plots/Heroin_funnel.pdf]

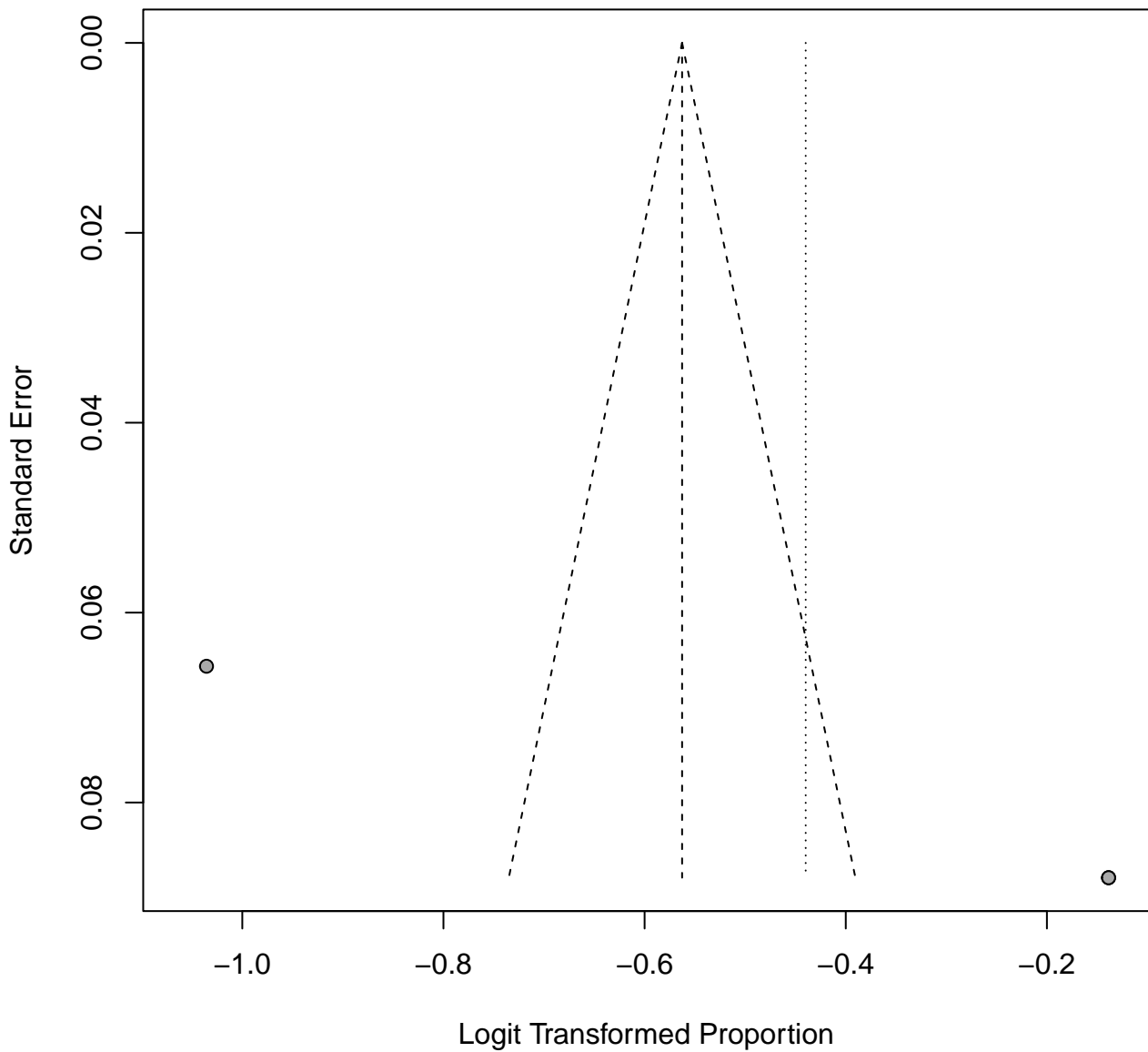

Supplement: S8 File — (ZIP) [file pgph.0004019.s008.zip › Supplementary File 4_Funnel Plots/Kolanut_funnel.pdf]

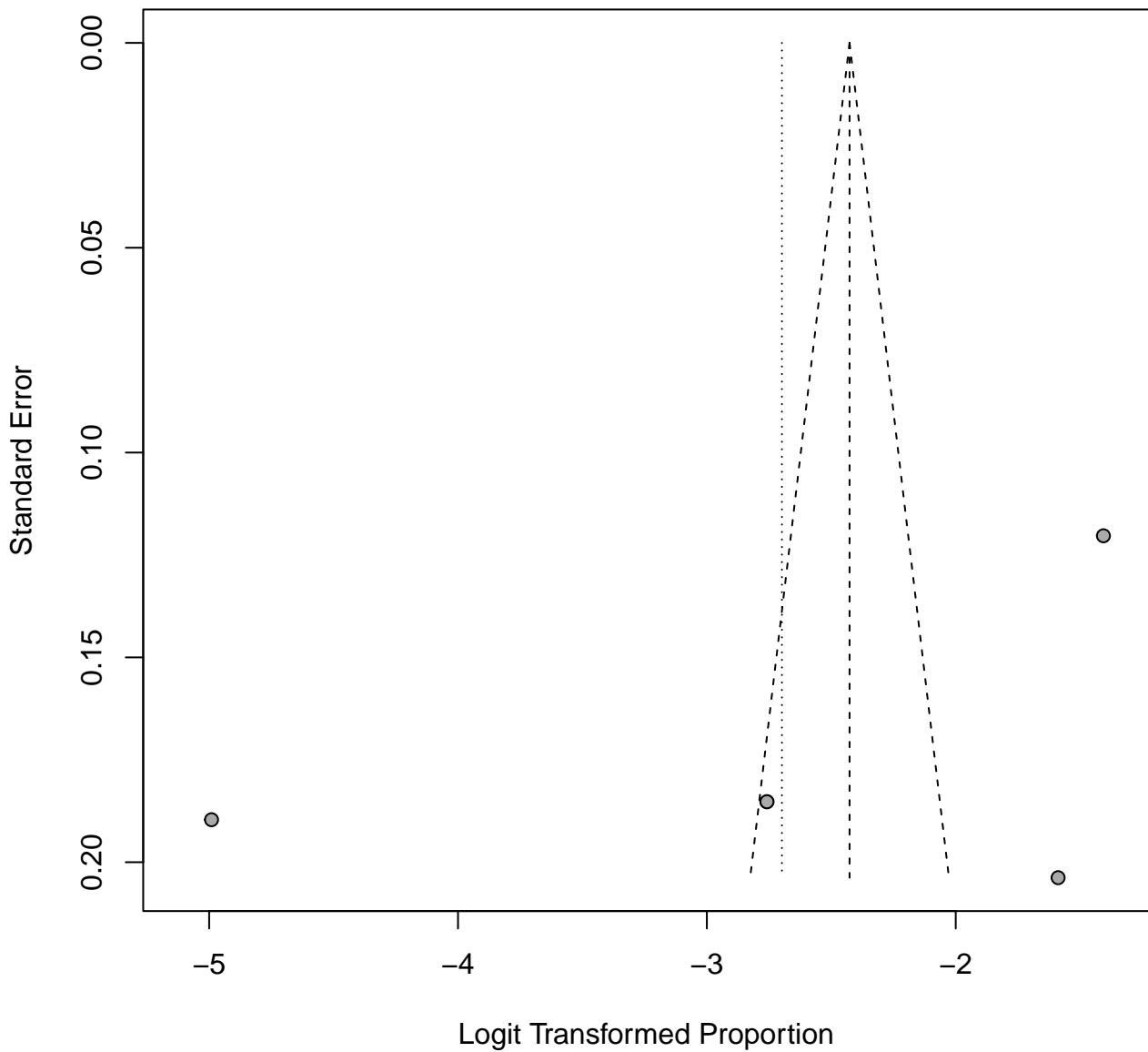

Supplement: S8 File — (ZIP) [file pgph.0004019.s008.zip › Supplementary File 4_Funnel Plots/Marijuana_funnel.pdf]

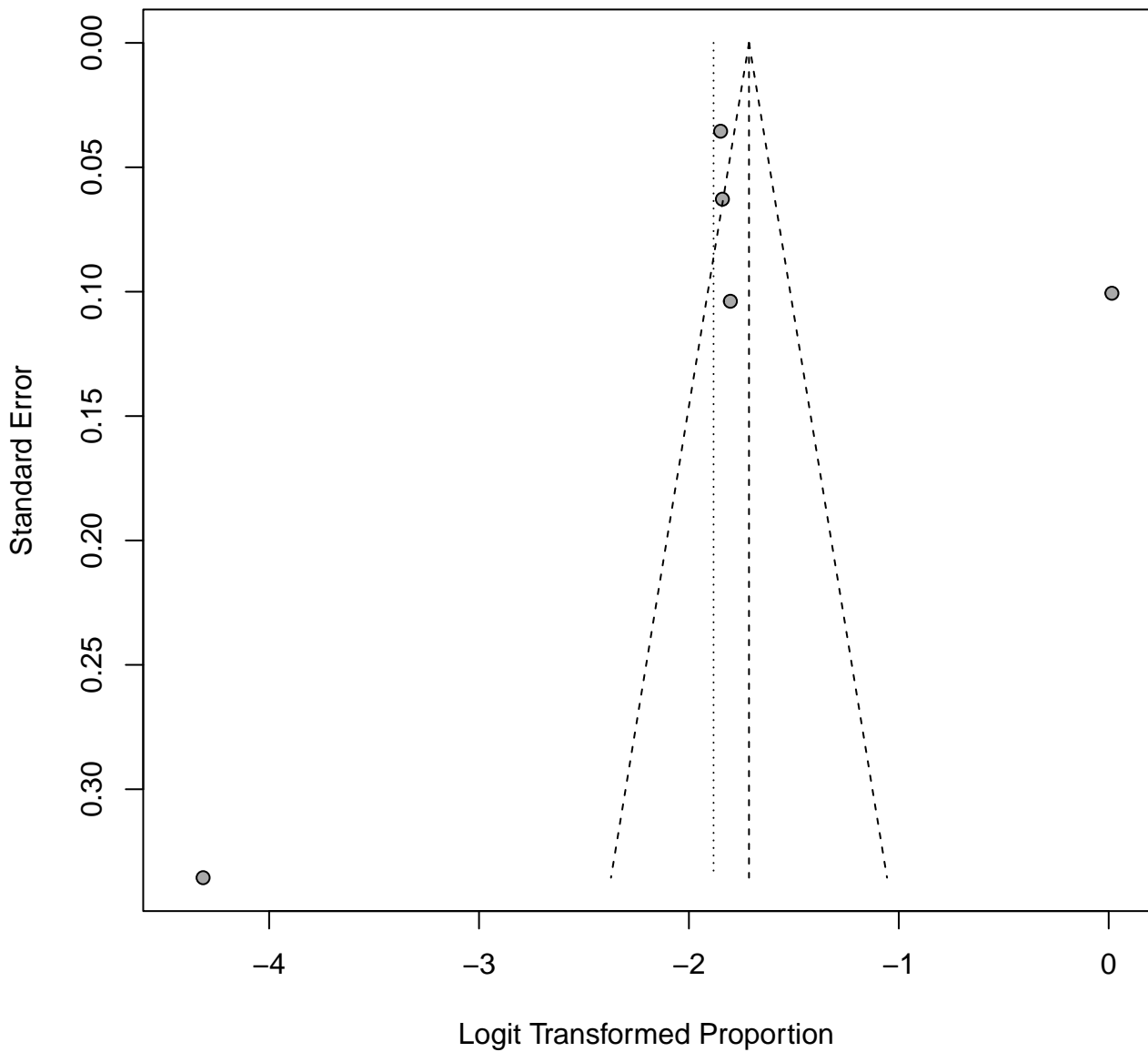

Supplement: S8 File — (ZIP) [file pgph.0004019.s008.zip › Supplementary File 4_Funnel Plots/Sedatives_funnel.pdf]

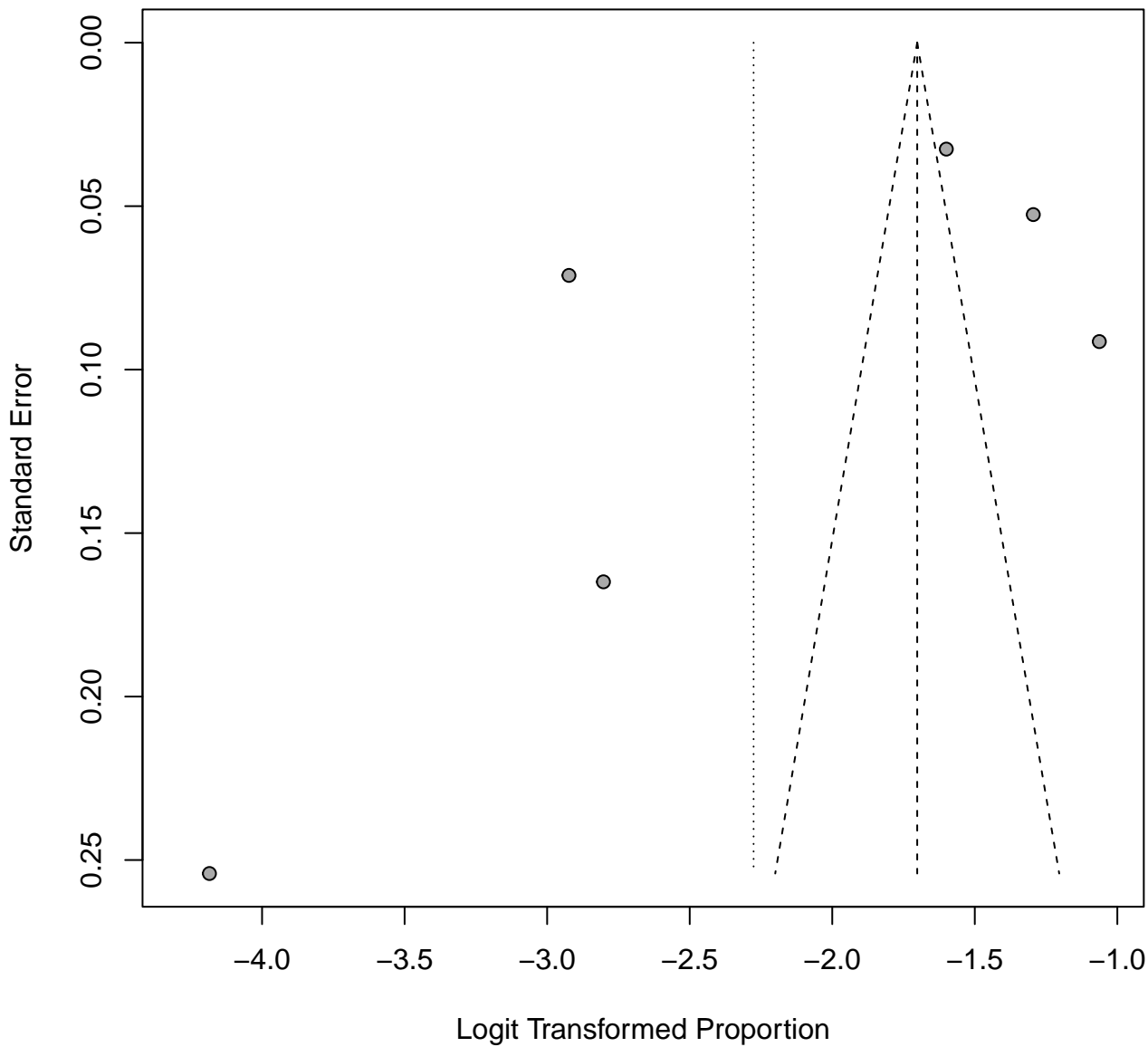

Supplement: S8 File — (ZIP) [file pgph.0004019.s008.zip › Supplementary File 4_Funnel Plots/Tobacco_funnel.pdf]

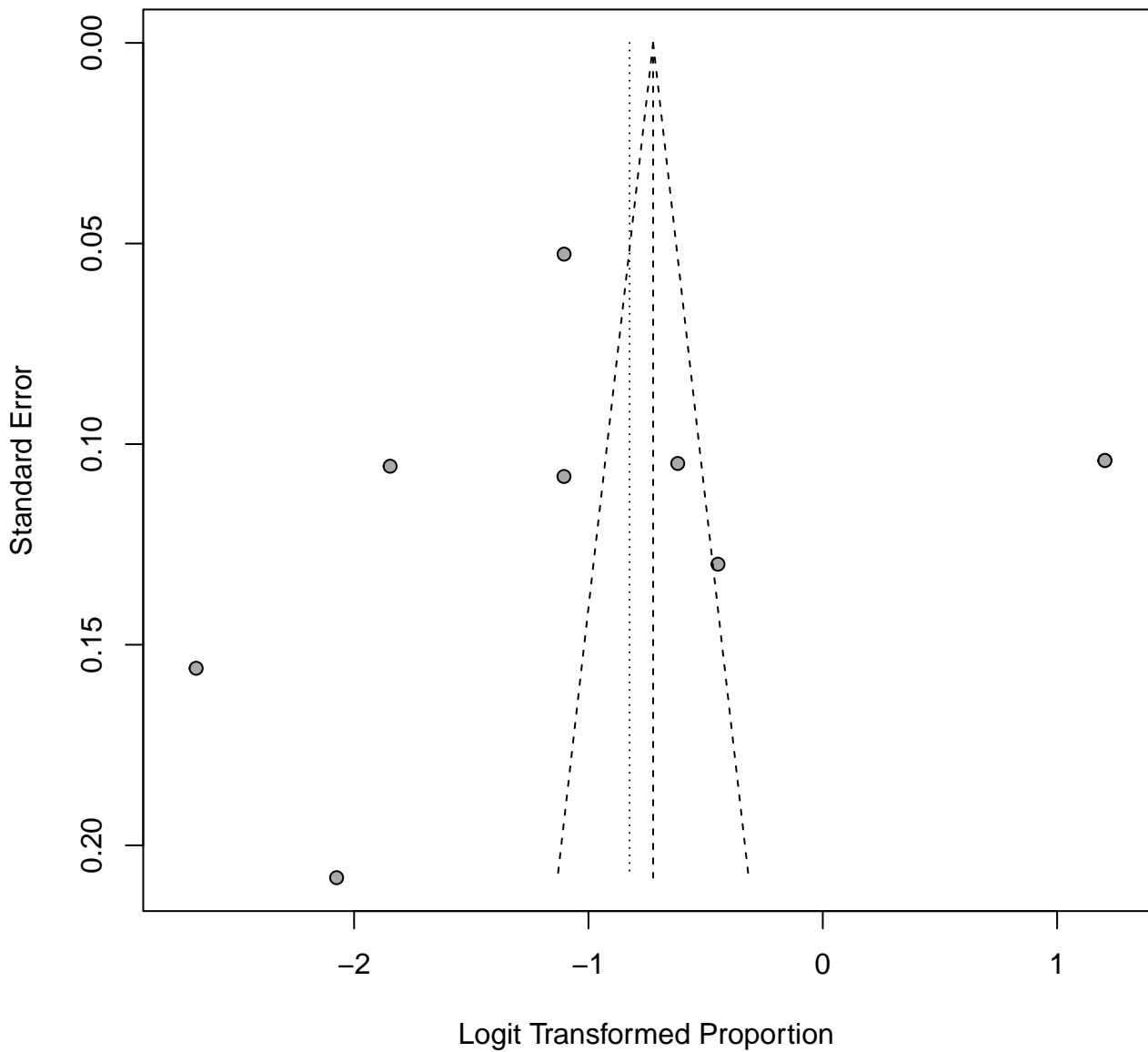

Supplement: S8 File — (ZIP) [file pgph.0004019.s008.zip › Supplementary File 4_Funnel Plots/Tremadol_funnel.pdf]
