# Supplementary material for: Prevalence and patterns of substance use in West Africa: A systematic review and meta-analysis
Source: PLOS Glob Public Health. 2024 Dec 31;4(12):e0004019. doi: 10.1371/journal.pgph.0004019 (PMC11687703; doi:10.1371/journal.pgph.0004019)
Supplement: Attachment — Submitted filename: Response to Reviewer 3.pdf [file pgph.0004019.s009.pdf]

Response to Reviewer 3

Thank you for your detailed review and insightful comments. We have carefully considered each point and made revisions to improve the clarity and accuracy of our manuscript. Below are our responses to your comments:

1. **Line 134: Studies Eligibility Criteria Comment:** Studies included individuals of all (should be 'ANY') age groups residing in West African countries. But many studies included in the analysis have specific populations (students, pregnant women, adolescents, etc.). Suggested to change the criteria to ANY.

   Response: Thank you for pointing out the need to refine the wording in the eligibility criteria. We have revised the text in line 134 from "all age groups" to "any age groups" to better reflect the study populations included in the analysis, such as students, pregnant women, and adolescents.

2. **Line 231: Alcohol emerged as the most frequently abused substance, followed closely by nicotine in the form of cigarettes. These findings highlight the widespread acceptance and availability of these substances. Comment:** It's a repeated sentence. It is suggested to provide a single table of pooled prevalence of various types of substance use.

   Response: Thank you for noting this repetition. The sentence in line 231 is part of the narrative synthesis based on the data from Table 3, which presents an overview of substances used in West Africa. While Figure 2 provides a summary of the pooled prevalence of alcohol use, the narrative offers additional context regarding the cultural acceptance and availability of substances such as alcohol and nicotine. To avoid redundancy, we have rephrased the sentence for better flow and to avoid repetition of information already provided in the table.

   Revised Text: *"Alcohol was identified as the most commonly abused substance, followed closely by nicotine, primarily in the form of cigarettes."*

3. **Line 235: The use of amphetamines and methamphetamines was also significant. Comment:** I cannot see this in results tables or figures.

   Response: Thank you for your observation. The mention of amphetamines and methamphetamines in line 235 originates from the narrative synthesis based on Table 3, which categorizes substances used in West Africa. While these substances were reported in some studies, they were not widespread enough across the included studies to feature in the pooled prevalence meta-analysis. However, their importance as emerging substances of abuse was noted in the narrative to highlight a growing trend. We have revised the text to better align with the information presented in Table 3.

   Revised Version: *"While not as commonly reported as other substances, the use of amphetamines and methamphetamines was noted in some studies, reflecting an emerging trend in stimulant abuse among certain populations."*

We hope these revisions address your concerns and contribute to the clarity and quality of the manuscript. Thank you once again for your valuable feedback.
